# 

**Published:** 1913-09

**Authors:** 


OFFICIAL ORGAN—State Society Social Hygiene and Eugenics.

Vol. XXIX SEPTEMBER, 1913 No. 3

### The "Red Ba[illegible]"

### TEXAS MEDICAL JOU[illegible]AL

Surgeon General, U. S. A.,
Washington, D. C.

ESTABLISHED JULY, 1885

PUBLISHED MONTHLY AT AUSTIN, TEXAS, BY

F. E. DANIEL, M. D., - - - Editor and Proprietor.

PRINTED BY THE VON BOECKMANN-JONES CO.

Subscription, $1.00 a Year, in Advance. - - - - Single Copies, 10 Cents

Entered at the Postoffice at Austin, Texas, as second class mail matter.

